# Supplementary figures and images for: Rsph4a is essential for the triplet radial spoke head assembly of the mouse motile cilia
Source: PLoS Genet. 2020 Mar 23;16(3):e1008664. doi: 10.1371/journal.pgen.1008664 (PMC7147805; doi:10.1371/journal.pgen.1008664)

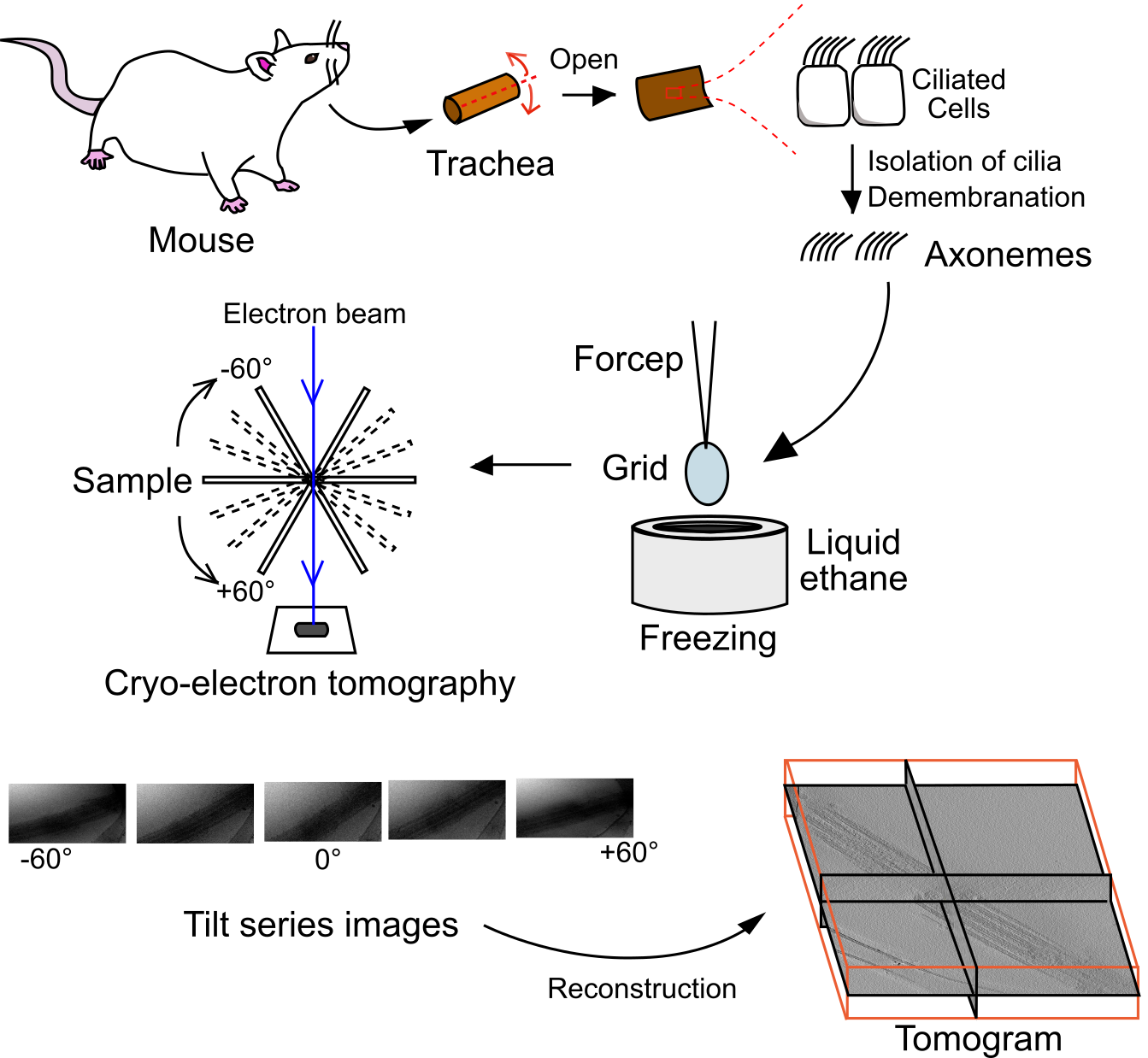

Supplement: S1 Fig — We dissected the mouse trachea and delicately rubbed it onto the wall of the tube to isolate cilia. Then, we collected the trachea cilia by ultracentrifugation, and the cilia were frozen in liquid ethane. Three-dimensional structures of the repeat unit of the axoneme are revealed by cryo-ET including cryoelectron microscope observation and subtomogram averaging (Methods). (TIF) [file pgen.1008664.s001.tif]

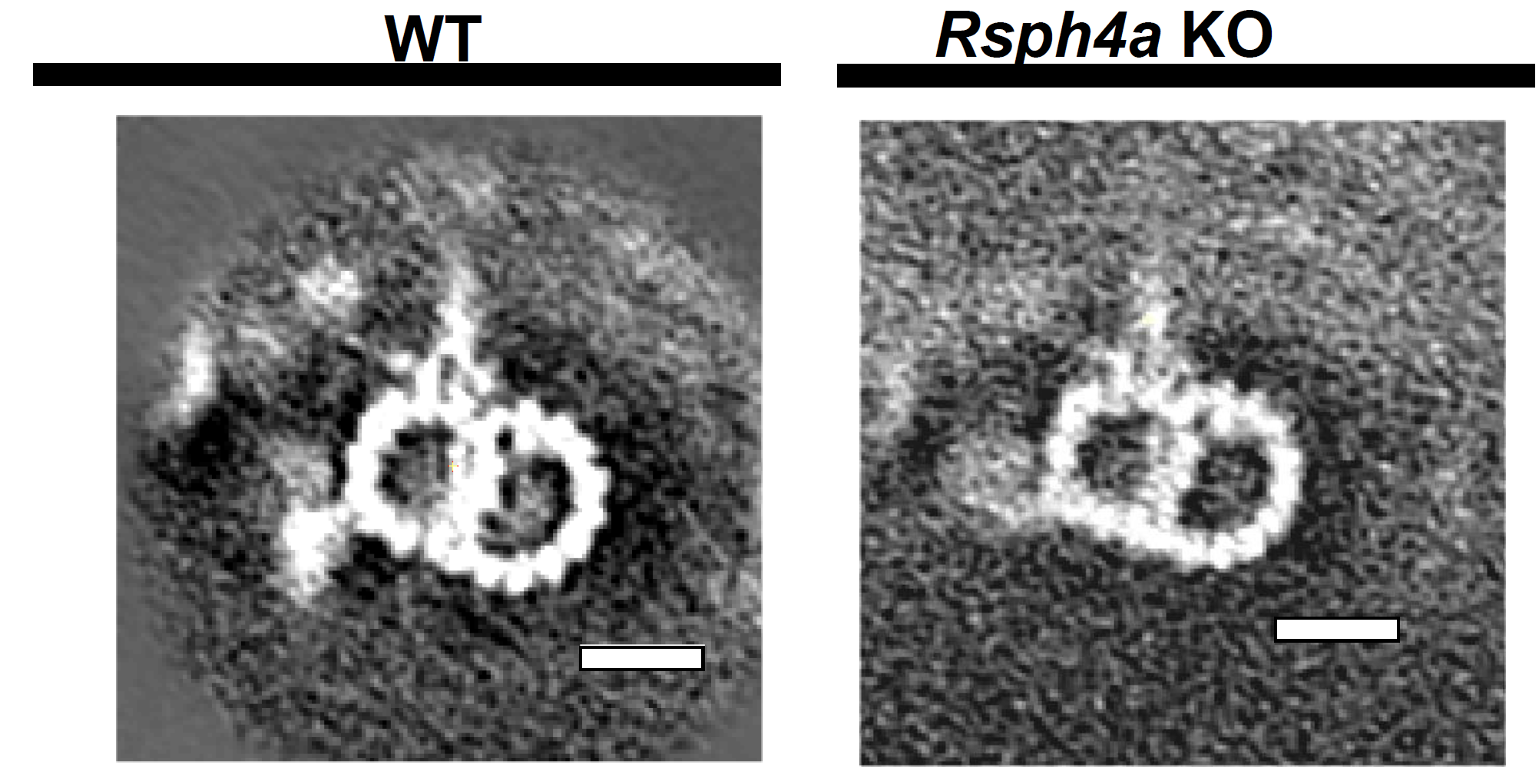

Supplement: S2 Fig — We show the tomographic slice of the trachea cilia in the wild type mice (left) and in the Rsph4a KO mice (right). Bars are 20 nm. (TIF) [file pgen.1008664.s002.tif]

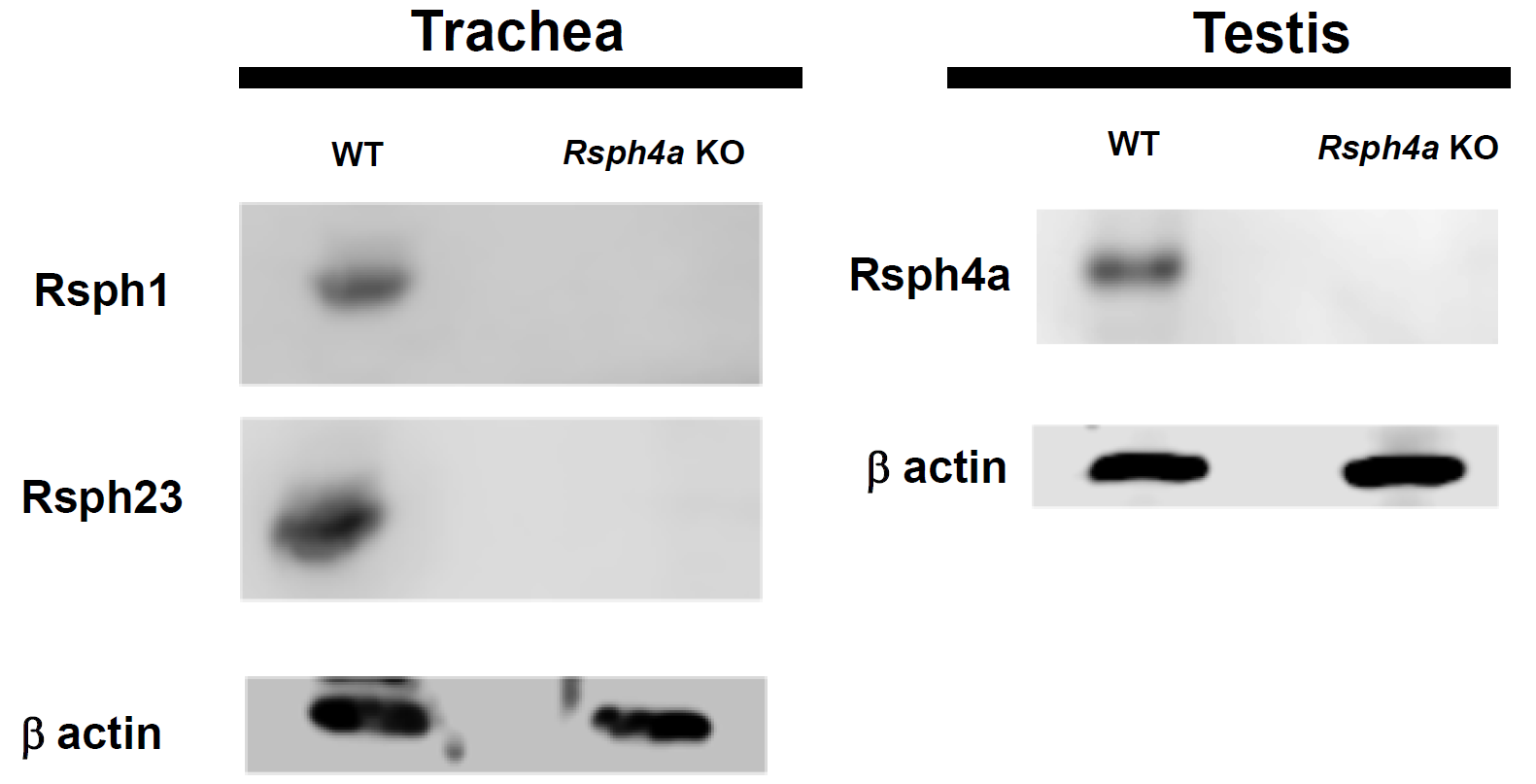

Supplement: S3 Fig — We show western blotting data of spoke head protein (Rsph4a, Rsph1), and neck/arch protein (Rsph23). Rsph1 and Rsph23 are reduced in the trachea of Rsph4a KO mice. (TIF) [file pgen.1008664.s003.tif]
