# Supplementary material for: Rsph4a is essential for the triplet radial spoke head assembly of the mouse motile cilia
Source: PLoS Genet. 2020 Mar 23;16(3):e1008664. doi: 10.1371/journal.pgen.1008664 (PMC7147805; doi:10.1371/journal.pgen.1008664)
Supplement: Attachment — Submitted filename: Response_KShinohara.pdf [file pgen.1008664.s011.pdf]

Response to the reviewers' comments

We are grateful to supportive and constructive comments from three reviewers. We have fully responded to the comments by performing additional experiments requested.

Reviewer #1

This manuscript by Yoke et al. investigates the loss of radial spoke protein RSPH4A in mouse motile cilia structure. Although this gene was previously implicated in human primary ciliary dyskinesia and mouse ciliary motility, this study uses a combination of cryoelectron tomography and immunofluorescence to uncover the role of RSPH4A in radial spoke assembly. In addition, high-speed video microscopy also reveals subtle but distinct differences between ciliated cell types in the mouse. These data are novel and important, the methods are appropriate, and the conclusions are generally justified. However, the manuscript would benefit from addressing a few concerns prior to publication:

MAJOR COMMENTS:

1. *FIGURE 1: Figure 1A is not discussed in the manuscript. What is the gross phenotype of these mice?*

Response: Thank you. As requested, we have described the explanation of Fig.1A. This is now mentioned in the text (page 4, "RESULTS" 1st paragraph, 3rd line).

2. *FIGURE 2: Figure 2D is not discussed in the manuscript.*

Response: Thank you. As requested, 8th we have described the explanation of Fig.2D. This is now mentioned in the text (page 5, "RESULTS" 1st paragraph, 8th line).

3. *FIGURES 5, 6, and 7: The authors state that RSPH9 and RSPH1 are "dramatically reduced" in all mutant ciliated cell types. However, while RSPH9 appears to be reduced, RSPH1 looks very similar to wild type in the tracheal epithelial and ependymal cells (Fig. 5P, 6P). Similarly, the authors state that RSPH23 is not present in cilia from any of the cell types, but it appears to be present in the trachea*

(Fig. 5V). How consistently are these proteins reduced in the mutant cells? Could fields that are more representative be shown in these figures? Quantitative western blotting could also potentially address the difference in protein levels that might be difficult to see by immunofluorescence.

Response: Thank you very much for this comment. This is absolutely correct. As pointed, localization of Rsph1 is retained in the ciliated cells in the trachea (Fig.5P) and the brain (Fig.6P) of *Rsph4a* KO mice in our immunofluorescence (IF) data. It is very difficult to quantify and compare the IF data between wild type and KO mice. Thus, we have carried out western blotting according to your suggestion (Fig.S3). In the trachea, we detect significant difference of protein level of Rsph1 between the wildtype and the *Rsph4a* KO mice, suggesting that level of Rsph1 is reduced in trachea ciliated cells of *Rsph4a* KO mice (Fig. S3). Although we cannot explain the exact reason of the difference between IF and western blotting, one possibility is that small amount of Rsph1 protein still remains in *Rsph4a* KO cells and this may be amplified by antibody reaction. Please note that we have carried out western blotting of Rsph4a using the testis tissue, because our antibody cannot detect clear signal band of Rsph4a protein in the trachea tissue.

In addition, on Rsph23, we have carried out western blotting. In the trachea, we can detect significant difference of protein level of Rsph23 between the wildtype and the *Rsph4a* KO mice as well as Rsph1 (Fig.S3). Therefore, we conclude that protein both level of Rsph1 and Rsph23 is reduced in the trachea cells of *Rsph4a* KO mice. This is now mentioned in the text (page 6, "RESULTS" 1st paragraph, 7th Line & 18th Line) and a new figure (Fig. S3).

4. *INTRODUCTION, 1st paragraph: The authors describe the ciliary motility defect in patients with mutations in RSPH1, RSPH4, and RSPH9, but it would be helpful if they also discussed the ultrastructural defects in the cilia from these patients, as that likely contributes to the abnormal beating patterns. In addition, human PCD patients have been reported with mutations in RSPH3 (Jeanson et al., 2015), which should be referenced.*

Response: Thank you for this comment. We have added the explanation of the

ultrastructural phenotype of the patients in the text (Page 3, "INTRODUCTION" 1st paragraph, 14th line). Further, we have now mentioned mutations of *RSPH3* and added the paper to the reference (Jeanson et al., 2015) (ref. 21).

5. *METHODS: Some important information is missing from the Methods Section. The following should be added:*

- *Generation of Rsph4a-/- mice: The genetic background of the mutant mice*
- *Immunofluorescence: The number of mice used for each experiment, the concentration or dilution factor for each primary antibody, the specific secondary antibodies*
- *Imaging of ciliary motion: The age and number of mice used for the experiment, the method of tissue collection and preparation*
- *Cryoelectron tomography: The age and number of mice used for the experiment*

Response: Thank you. We have added information on (i) the genetic background of the mutant mice (Page 7, "Generation of Rsph4a–/– mice"), (ii) the detail of immunofluorescence (Page 8, "Immunofluorescence"), (iii) the detail of imaging of ciliary motion (Page 8, "Imaging of ciliary motion"), and (iv) the detail of cryoelectron tomography (Page 9, "Cryoelectron tomography of mouse trachea cilia"), and (v) the detail of western blotting (Page 8, "Western blotting").

MINOR COMMENTS:

1. *INTRODUCTION, 1st paragraph: The authors state that "multiple motile cilia exist in the trachea, brain/ependymal, oviduct, inner ear, nasal, testis, and so on." The phrase "and so on" should be specified, as some readers may not know the exact locations of motile cilia in the body.*

Response: Thank you. This is correct. To prevent misleading, we have deleted the phrase of "and so on" (Page 3, "INTRODUCTION", 1st paragraph, 5th Line).

2. *DISCUSSION, 2nd paragraph: The authors state that "the stability of the neck/arch is most likely different among species." This is an important point, but is it possible*

that assembly of the neck/arch could be different as well?

Response: Thank you very much for this comment. This is absolutely important point. Our data suggests that the neck/arch is disrupted in the trachea cilia of *Rsph4a* KO mice. To address this issue, we should compare our cryo-ET structure with cryo-ET structure of trachea cilia in Rsph4a-deficient human patients or the other vertebrate. Unfortunately, we have not obtained any cryo-ET data of human or the other vertebrate yet, we have better change the tone of the sentence at this time. We have changed the phrase of "is most likely" to "may be" in the sentence (Page 7, "Discussion" 2nd paragraph, 10th Line)

3. METHODS, Generation of Rsph4a-/- mice: The design of the targeting vector needs an appropriately formatted reference.

Response: Thank you. The design of the targeting vector is shown in supplementary figure of our previous paper (Fig.S4 in the paper [Ref.30, Shinohara etal 2015]). We have now mentioned this point in the text (Page 8, "Generation of Rsph4a–/– mice", 1st line).

<u>Reviewer #2</u>

Recent cryoelectron tomography data reveal three types of radial spokes (RS1, RS2, and RS3) in the 96 nm axoneme repeat unit; however, the molecular composition of the third radial spoke, RS3 is unknown. Here, the authors describe that Primary Ciliary Dyskinesia protein Rsph4a plays critical role in the radial spoke head assembly not only of RS1 and RS2 but also of RS3. Examination of the cryoelectron tomography structure and the immunofluorescence analyses of wild type and Rsph4a-deficient mutant mice led them to conclude that Rsph4a is a generic spoke head protein of the triplet radial spoke in mouse motile cilia.

The manuscript is nicely written and well understandable.

The main and novel claim, that Rsph4a plays critical role in the radial spoke head assembly not only of RS1 and RS2 but also of RS3 is significant both for the field of basic cilia biology and the diagnostic field, because it provides additional information

about cilia structure and an explanation of the diverse phenotype of patients with Primary Ciliary Dyskinesia (PCD) caused by radial spoke defects.
This finding is nicely demonstrated and supported by cryo-EM data of trachea of WT and Rsph4 mutant mice. However, there are major points to consider prior to publication and additional information is needed prior to publication:

1. Page 5: "however, the ciliary localization of Rsph9 and Rsph1 was dramatically reduced in the tissues (Fig. 5 J-L and P-R, Fig. 6 J-L 6 and P-R, Fig. 7 J-L and P-R)" Please provide figures with higher quality. Especially acetylated tubulin appears to be overexposed.

a) Please quantify reduction compared to control; how do you explain the reduction, taking into account your cryo-EM results of trachea that suggest complete loss of at least the head, neck and arch?

Response: Thank you very much for this comment. This is absolutely important. At first, we have submitted again images of acetylated tubulin with shorter exposure time accordingly (Fig.5-7). Next, we have tried to quantify intensity of immunofluorescence (IF) data of Rsph9 and Rsph1 and compare the wildtype with the *Rsph4a* KO. However, it is very difficult for us to evaluate because there is no linear relationship between intensity of IF images and amount of protein. Thus, alternatively, we have carried out western blotting using the trachea and the testis tissue. In result, we have observed significant differences of Rsph1 between wild type (Band intensity = 131,000) and Rsph4a KO mouse (Band intensity = 5,180) in the trachea tissue (Fig. S3). The IF data of the trachea show some remaining intensity of Rsph1 in cilia of *Rsph4a* KO mice (Fig.5P). Although we cannot explain the exact reason of the difference between immunofluorescence and western blotting, one possibility is that small amount of Rsph1 protein still remains in *Rsph4a* KO cells and this may be amplified by antibody. Please note that we have carried out western blotting of Rsph4a using the testis tissue, because our antibody cannot detect clear signal band of Rsph4a protein in the trachea tissue.

On Rsph9, we believe that an apparent difference of Rsph9 is observed between wildtype and Rsph4a KO in our IF data (Fig.5G&J, Fig.6G&J, Fig.7G&J). These revisions are now mentioned in the text (Page 6, "RESULTS" 1st paragraph, 5th line) and Figures (Fig.5-7 & a new figure (Fig. S3)).

b) *Do you see differences in cryo-EM results between tracheal cilia, ependymal cilia, oviduct? Differences in your IF result suggest so. If not already done, cryo-EM on those additional cilia would improve your data and conclusions.*

Response: We have not obtained cryo-EM results of ependymal and oviduct cilia yet. We are trying to determine cryoEM structure of mouse oviduct cilia. Unfortunately, however, number of isolated cilia is not sufficient to obtain 3D structure (bottom Fig), thus this must be our future work. On the ependymal tissue, we have not succeeded to isolate cilia. This is technically tough at this time.

CryoET of mouse oviduct cilia (Preliminary)

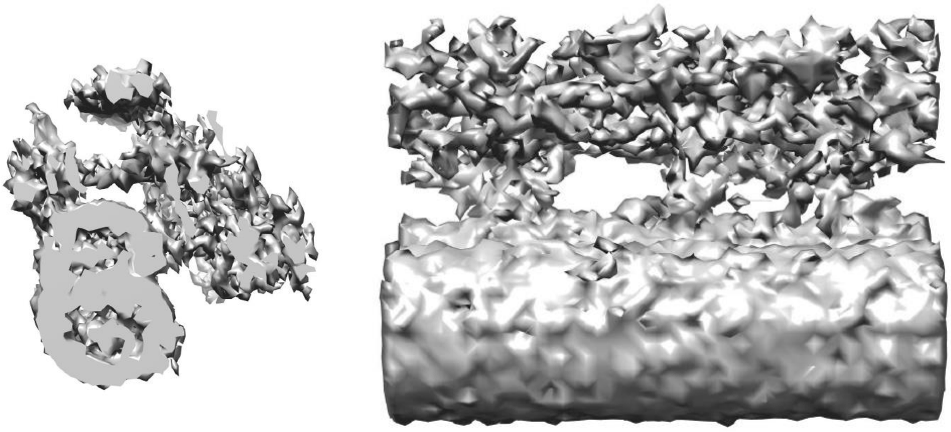

c) *How do you explain differences in localization of especially Rsph1 and Rsph23 in trachea, ependymal cells, oviduct? Especially the normal appearing Rsph1 localization in trachea and ependyma of Rsph4 mutant mice?*

Response: Thank you for this comment. As you point out, our IF data show Rsph1 localization seems normal in the trachea and the ependymal cells of Rsph4a KO mice. To clarify protein level of Rsph1 and Rsph23, we have carried out western blotting using the trachea tissue. In result, we have observed significant difference of level of both Rsph1 and Rsph23 between the wild type and the Rsph4a KO mouse (Fig.S3). This suggests that Rsph1 and Rsph23 decrease in the Rsph4a KO cells in the trachea. We conclude that the spoke head proteins (Rsph1, 9) and the neck protein (Rsph23) are affected in the trachea cells of Rsph4a KO mice. This is now mentioned in the text

(Page 6, "RESULTS" 1st paragraph, 5th line& 17th line) and a new figure (Fig. S3).

d) Rsph9 staining in Figure 7 appears to be basal body staining, please comment

Response: Thank you for this comment. In Fig.7L, Rsph9 seems to stay at the basal body in the oviduct of Rsph4a KO mice. But, we are not sure this is indeed basal body localization since we do not see basal body marker.

2. Page 6: "Conversely, Rsph23 was not localized in the axoneme of the motile cilia in the trachea, ependymal tissues, and the oviduct tissues in Rsph4a KO mice (Fig. 5 V-X, Fig. 6 V-X, Fig. 7 V-X)": Rsph23 is detected in cilia of Rsph4 KO trachea as shown in Figure 5. Please correct and comment.

Response: Thank you very much for this comment. This is absolutely correct. We have corrected the sentence: "In the trachea, weak staining of Rsph23 retained in the ciliated cells of Rsph4a KO mice (Fig. 5 V-X). To validate the difference of level of protein, we carried out western blotting using the trachea tissues and we observed significant difference of protein level of Rsph23 between the wildtype and Rsph4a KO mice (Fig. S3). The western blotting data indicate that Rsph23 was reduced in the trachea cells of Rsph4a KO mice (Fig. S3)." (Page 6, 1st paragraph,17th line). From the western blotting data (a new figure: Fig.S3), we conclude that Rsph23 is reduced in the trachea cells of Rsph4a KO mice and that the spoke head and the neck/arch are disrupted in the absence of Rsph4a in the three types of motile ciliated mouse tissues.

3. *Materials and Methods, Imaging of ciliary motion: Information about sperm has not been provided, but mentioned in M&M. Please correct or include data.*

Response: Thank you for this comment. This is absolutely correct. We have motion data of sperm in Rsph4a KO mice, but the number of example is not sufficient (N = 2 cells). We have deleted information on the sperm in M&M.

4. *Supplementary videos 1,5,6 appear to be different format and cannot be assessed.*

Response: Thank you. We have revised and unified the format of Video1, Video5, and Video6: (the number of frames and the speed of movie).

5. *Please use proper nomenclature for proteins in mouse*

Response: Thank you. We have corrected “Rsph4a KO mice” to italic “*Rsph4a* KO mice.

Reviewer #3

The functional relationships between the structural organization of the cilia and their beating behavior is a fascinating biological question. Recent advances in cryo-electron tomography observations allowed major breakthroughs in understanding the architecture of the different components involved in ciliary motility.

In this manuscript, the authors analyze the role of the radial spoke protein Rsph4 in mouse multiple ciliated epithelia. In all three type of Rsph4-/- multiple ciliated epithelia, ciliary motility is affected, with striking differences between tissues. For the first time the authors describe the ultrastructural organization of the radial spokes in mouse ciliated airways. They show that like in other vertebrates, 3 different radial spokes can be observed in the 96 nm axonemal repeats. Interestingly, they observed marked differences with humans in the architecture of the spoke head and in the connections between Radial spokes 2 and 3. They also show that Rsph4a is, a key component required for head formation of the three types of radial spokes. Last the authors show that Rsph4 is, like in humans, required for the assembly of Rsph1, Rsph9 at radial spokes but also, unlike in humans, of Rsph23 required for neck/arch assembly.
This manuscript thus highlights evolutionary divergences between species and pave the way to understand the origin of the diversity of cilia motile properties.

I only have a few minor concerns regarding the presentation of the data:

1. *-Could the authors present at least one or two examples of the averaged tomographic images and not just the surface rendering of it?*

Response: Thank you for this comment. We have added the averaged tomographic images in Fig.S2 accordingly. This is now mentioned in the text (Page 5, "Rsph4a is essential for triplet radial spoke head assembly in the mouse motile cilia" 1st paragraph, 3rd line).

2. *-I am not sure to understand the description of the differences between human and mouse. Human radial spoke heads were compared to pairs of ice blade in Lin et al 2014, and on panel 2B mouse radial spoke heads do not show such parallel "blades"? The text and conclusions are confusing compared to the images and with the initial description of human radial spokes in Lin et al.*

Response: Thank you very much. As you point out, our cryo-ET data of spoke head does not show clear parallel blades (Fig.2B). To prevent misleading, we have corrected and deleted the sentence of "In RS2 and RS3, spoke heads show an ice skate blade-like morphology (Fig. 2 B, Fig. 3A, B), which is observed in RS1 and RS2 of the human respiratory cilia [17]". (Page 5, "Cryoelectron tomography revealed the ultrastructure of the mouse motile cilia" 1st paragraph, 13th line).

3. *-The movie of control oviduct cilia is hard to compare to the rsph4 mutant ones, maybe another movie could help to better see the differences in motility?*

Response: Thank you. We have now presented another typical movie of oviduct cilia of the wildtype mouse (New Video 5).
